# Influence of the built environment on taxi travel demand based on the optimal spatial analysis unit

**DOI:** 10.1371/journal.pone.0292363

**Published:** 2023-10-03

**Authors:** Yaxin Duan, Changwei Yuan, Xinhua Mao, Jiannan Zhao, Ningyuan Ma

**Affiliations:** 1 College of Transportation Engineering, Chang’an University, Xi’an, China; 2 Engineering Research Center of Highway Infrastructure Digitalization, Ministry of Education, Chang’an University, Xi’an, China; University of Glasgow College of Medical Veterinary and Life Sciences, UNITED KINGDOM

## Abstract

When discussing the influence of the built environment on taxi travel demand, few studies have considered the effect of the modifiable areal unit problem (MAUP) or the influence of the “5D” dimensions of the built environment (It refers to the consideration of the built environment from five dimensions of density, diversity, design, destination accessibility and distance to transit.) on taxi travel demand. Moreover, discussion of the nonlinear and linear relationships between taxi demand and environment variables is also lacking. To address these gaps, we constructed a “5D” dimension index system of built environment variables. The influence of the MAUP on the model results was discussed using the optimal parameter-based geographical detector (OPGD) model, and the optimal spatial analysis unit was selected. The OPGD and multiscale geographically weighted regression (MGWR) models were used to reveal the influence of different dimensions of the built environment on taxi travel demand from global and local perspectives, respectively. Finally, the central urban area of Xi’an was analyzed as an example. The results show the following: (1) Most built environment variables are sensitive to the influence of MAUP. (2) It is better to divide the space into regular hexagons than squares, and the optimal spatial analysis unit in this study is a regular hexagon grid with sides of 900m. (3) From a global perspective, the distance to the city center, commercial residence POI density, transportation facility POI density, and population density have the greatest influence on the demand for taxi travel. (4) From a local perspective, the MGWR model considering spatial heterogeneity and scale differences is superior to the GWR model, and the influence of built environment variables exhibited spatial heterogeneity. The proposed optimal spatial analysis unit can provide a basis for taxi demand forecasting and scheduling. This study provides a reference for urban planners and traffic managers to offer optimization strategies related to the built environment, promote healthy development of the taxi industry, and solve the problems of the urban transportation system.

## 1 Introduction

With increasing urbanization, the urban population, number of motor vehicles, and infrastructure are also increasing rapidly. This series of changes has led to the reorganization and transformation of urban spaces, producing large and complex urban systems. As a result of urban space changes, the built environment is now an important carrier affecting the population’s travel behavior. Therefore, many scholars have advocated changing the built environment through land-use planning and transportation policies to guide travel behaviors, improve the urban traffic environment, and alleviate traffic congestion [1]. Taxis are one of the most critical transportation modes for urban residents and play an indispensable role in travel behavior owing to their flexibility and convenience. The built environment also influences them. In recent years, scholars have also shifted their research focus to the relationship between the built environment and taxi travel behavior. These studies have focused on the mechanism by which the built environment influences behavioral factors, such as the taxi travel distance, travel intensity, and travel time [2–4]. The results have confirmed that the built environment influences taxi travel behavior to varying extents.

The demand for taxi travel is an essential behavioral factor in taxi travel behavior that is coupled with the non-static and varying qualities of built environment elements. As a result, the uneven and complex distribution of built environment elements in urban spaces directly affects the spatiotemporal distribution of residents’ taxi travel demand. In addition, drivers have an inadequate grasp of high-demand areas, which often leads to difficulties in taking taxis and long wait times, which are not conducive to urban development. Therefore, some scholars have begun to pay attention to the influence of built environment variables such as population, land use, and road design on taxi travel demand [5–7]. These studies aim to deeply understand the connection between the built environment and taxi travel demand. Thus, these studies can provide a scientific basis for taxi demand prediction and scheduling. In addition, these studies are important for management services in the taxi industry and for promoting the virtuous cycle of urban transportation.

However, when constructing a model of the influence of the built environment on taxi travel demand, few studies have considered (or have considered insufficiently) the differences in the results caused by the modifiable areal unit problem (MAUP) when aggregating data. The MAUP refers to a phenomenon in which analysis results will vary depending on the definition of the primary research units (as first proposed by Openshaw in 1984 [8]). These differences in definitions mainly involve scale and zoning effects [9, 10]. The scale effect refers to changes in the statistical results of aggregated spatial data that occur when the size of the research unit changes. The zoning effect refers to differences in the statistical results caused by the aggregation of spatial data using different zoning schemes for a fixed research unit size. Some studies have also shown that the MAUP is an essential fundamental issue in many traffic problem studies [11]. For example, Zhao et al. [12] studied the impact of the built environment on online car-hailing travel intensity and found that as the analysis scale increased, the effect of proximity to public transportation on online car-hailing travel intensity increased; however, its effect was not significant at the remaining analysis scales. Therefore, the MAUP cannot be ignored when constructing a model of the influence of built environment on taxi travel demand, and a suitable spatial analysis unit is a prerequisite for such research.

This paper aims to explore the relationship between the built environment and taxi travel demand. Based on point of interest (POI), population, and road network data, the built environment variables were reasonably quantified. The optimal discretization of built environment variables under the MAUP effect was determined through the optimal parameters-based geographical detector (OPGD) model. Then, according to the optimal parameter combination results, the relationship between the built environment and taxi travel demand was analyzed under the MAUP effect, and the optimal spatial analysis unit for the influence of the built environment on taxi travel demand was determined. On this basis, while considering the nonlinear and linear relationships between the built environment and taxi travel demand, the influence of the built environment on taxi travel demand was explored by cross-using the OPGD and multiscale geographically weighted regression (MGWR) models.

The potential academic contributions of this paper are as follows. (1) The optimal discrete parameter combination of the built environment variables is determined based on the OPGD model. This solves the lack of accurate quantitative evaluations of discretization methods and classification numbers when discretizing continuous variables in the OPGD model. (2) At the same time, the optimal scale and zoning scheme for aggregating the built environment and taxi travel demand data are determined using the OPGD model. The selection of spatial unit granularity for this type of problem is thus realized. (3) A “5D” dimension index system of the built environment is constructed to explore its influence on taxi travel demand. This provides a scientific basis for quantifying each index. (4) The OPGD and the MGWR models are cross-used. This method can not only reveal the degree of influence of major built environment variables on taxi travel demand from a global perspective but also reflects the spatial heterogeneity of the influence of the built environment on taxi travel demand from a local perspective. This provides a method for studying the influence of the built environment on taxi travel demand. The reasonable determination of the spatial analysis unit in this study can facilitate a better understanding of the influence of the built environment on taxi travel demand and improve the reliability of the results. At the same time, it also provides a meaningful method for reasonably integrating environmental and transportation policies to guide taxi travel and alleviate problems such as taxi-taking difficulties.

The remainder of this paper is organized as follows. Section 2 summarizes the related work. Section 3 introduces the main research methods used in this paper, including the method for determining the optimal spatial analysis unit, OPGD model, methods for testing the spatial autocorrelation and multicollinearity, and the MGWR model. Section 4 outlines the study area and data, including the study area and division, data sources, and processing. Section 5 presents an analysis and discussion of the results. Section 6 summarizes the principal conclusions, limitations, and future work.

## 2 Literature review

The determination of spatial units is the premise and foundation of spatial analysis. The size and shape of the spatial analysis units determine the amount of data to be included, resulting in differences in the data analysis results [13, 14]. Previously, when analyzing the influence of the built environment on taxi travel demand, the research area was often divided into a single space type using the crowd sampling method of “people for their use.” For example, the study area would be divided into a traffic analysis zone (TAZ) [15], census area [16], and grid [17], neglecting the impact of the MAUP on the data aggregation and modeling results. However, scholars have recently begun to focus on this issue. For example, Wang et al. [19] divided a study area into different grid scales and calculated the 90% quantile of built environment variables under different grids using the OPGD model. The 90% quantiles of the built environment variables were found to differ at different grid scales. The scale corresponding to the maximum value was selected as the optimal scale, and the impact of the built environment on ride-hailing travel demand was discussed. However, this study only considered the scale effect in the MAUP and ignored the other substantial zoning effect. Moreover, Cheng et al. [18] divided a study area into two spatial units, TAZ and 1 km × 1 km grids, to study the impact of the built environment, population distribution, and road network structure on taxi travel demand at night. The results showed that the R-squared value of the TAZ-based spatial Durbin model (SDM) was slightly lower than that of the grid-based SDM model; however, the Log-likelihood, Akaike information criterion (AIC), and Bayesian information criterion (BIC) of the former were significantly lower than those of the latter. Finally, the TAZ was selected as the spatial analysis unit. However, this study considered only the zoning effect and ignored the scale effect. Of course, some scholars have considered both the scale effect and zoning effect in the MAUP. Wang et al. [19] divided a study area into TAZ, Thiessen polygons, community units, and 300-1000m (100m interval) grids to explore the impact of the built environment on network passenger volume. The results showed that the road density exhibited a significant spatial clustering distribution in the community units, TAZ, and Thiessen polygons. In contrast, the bus station density exhibited a significant spatial clustering distribution under the 400m grid, 1000m grid, and community units. Gao et al. [20] divided the study area into three partition types: administrative partition, hexagonal grids, and square grids. Spatial units with basically the same corresponding scale were generated according to the street, community, and traffic district scales in the administrative partition, resulting in a total of nine partition schemes. The results showed that the influence of commercial land on taxi commuting demand decreased with an increase in spatial scale. Other studies that considered both the scale and zoning effects of the MAUP have explored the relationship between the built environment and shared bike travel [21] and the built environment and traffic system state [22]. Therefore, the MAUP should be fully considered when investigating the relationship between the built environment and taxi travel demand, which will inevitably affect the modeling results.

Built environment factors that affect taxi travel demand are complex. The built environment was initially constituted by the “3D” dimensions proposed by Cervero and Kockelman [23]: density (such as population density and the density of POIs), diversity (such as the mixed degree of land use), and design (such as the road network density and intersection density). Subsequently, this framework was expanded to the “5D” dimensions [24], which added the characteristics of destination accessibility (such as the distance to the city center and distance to CBD) and distance to transit (such as the distances to bus and subway stations). Currently, this framework has developed into the “7D” dimensions [25], adding demand management and demographic characteristics. However, while these two indicators are closely related to the built environment, they do not directly describe it [26]. The different dimensions of the built environment have led scholars to adopt different built environment variables in their research. For example, based on a review of 29 studies on public transportation and the built environment, Liu et al. [27] found that although many studies were consistent with the ‘5Ds,’ most did not include comprehensive coverage of all domains of the ‘5Ds;’ moreover, some variables used in these studies were inconsistent with the ‘5Ds.’ Chen et al. [28] quantified the built environment using various land-use variables to investigate the relationship between the built environment and taxi travel demand. However, the authors did not divide the dimensions of these variables. Zhu et al. [29] quantified the built environment in terms of road network density, bus coverage, subway coverage, and other variables. They did not explicitly propose the dimension concept of the built environment. Ni and Chen [30] also reported similar situation: only the variables of the built environment were quantified, and these variables were not divided into relevant dimensions. In addition, Xie et al. [31] divided built environment variables into four dimensions: density, design, diversity, and destination accessibility. However, these were not considered comprehensively. In addition, although Zhu et al. [32] mentioned the concept of 5Ds, only built environment variables from the four dimensions of density, design, distance to transit, and diversity were selected. These studies indicate that built environment factors are complex and variable. The mechanism by which the built environment influences taxi travel demand is also complex and needs to be strengthened and further explored.

When exploring the relationship between the built environment and taxi travel demand, some scholars have typically assumed a linear relationship between the built environment and taxi travel demand, and impact models considering spatial correlation and heterogeneity have been established, such as the SDM [33], spatial error model (SEM) [1], and geographically weighted regression (GWR) model [34]. Because all of the independent variables in the spatially heterogeneous GWR model have the same bandwidth, and the changes in the relationships between independent and dependent variables at different spatial action scales are ignored, the MGWR model has been developed. However, studies using this model to explore the impact of the built environment on taxi travel demand are rare [32]. This type of research can not only fit the relationship between the built environment and taxi travel demand but also analyze the spatial heterogeneity of the built environment effect at small scales. However, owing to the complexity and changeability of travel purposes and the limitations of urban activity spaces, taxi travel demand sometimes varies linearly with the built environment. Therefore, some scholars have proposed that nonlinear relationships should also be considered [35]. This research is generally conducted by introducing machine learning methods (such as the gradient boosting decision tree (GBDT) [36] and random forest (RF) models [37]). However, these studies have largely focused on the nonlinear relationship between the built environment and shared-bike usage [38–42], bus travel [43, 44], carpool usage [45, 46], and trail transit travel [47]. Research on the nonlinear relationship between the built environment and taxi travel demand remains lacking. Simultaneously considering the nonlinear and linear relationships between the built environment and taxi travel demand can allow for a more accurate exploration of the correlation between the two and determination of the hidden complex relationship.

In summary, previous studies have rarely considered the MAUP in the process of data aggregation and modeling when discussing the mechanism by which the built environment influences taxi travel demand. When constructing a built environment index system, the influence of the “5D” dimensions of built environment variables on taxi travel demand has rarely been considered. Regarding research methods, the nonlinear and linear relationships between the built environment and taxi travel demand have rarely been recognized. To solve these problems, this paper considered both the scale and zoning effects in the MAUP and divided the study area into multiple spatial units. Based on multi-source data and the characteristics of each dimension of the built environment, a “5D” dimension index system of built environment variables was constructed. Referring to the method reported by Wang et al. for selecting the optimal spatial analysis unit using the OPGD model [48], a more reasonable spatial unit division scheme was determined. The nonlinear and linear relationships between the built environment and taxi travel demand were thoroughly discussed by cross-using the OPGD and MGWR models [32]. Based on these existing methods, a more detailed and comprehensive analysis was realized than in previous studies.

## 3 Methods

**Fig 1:**
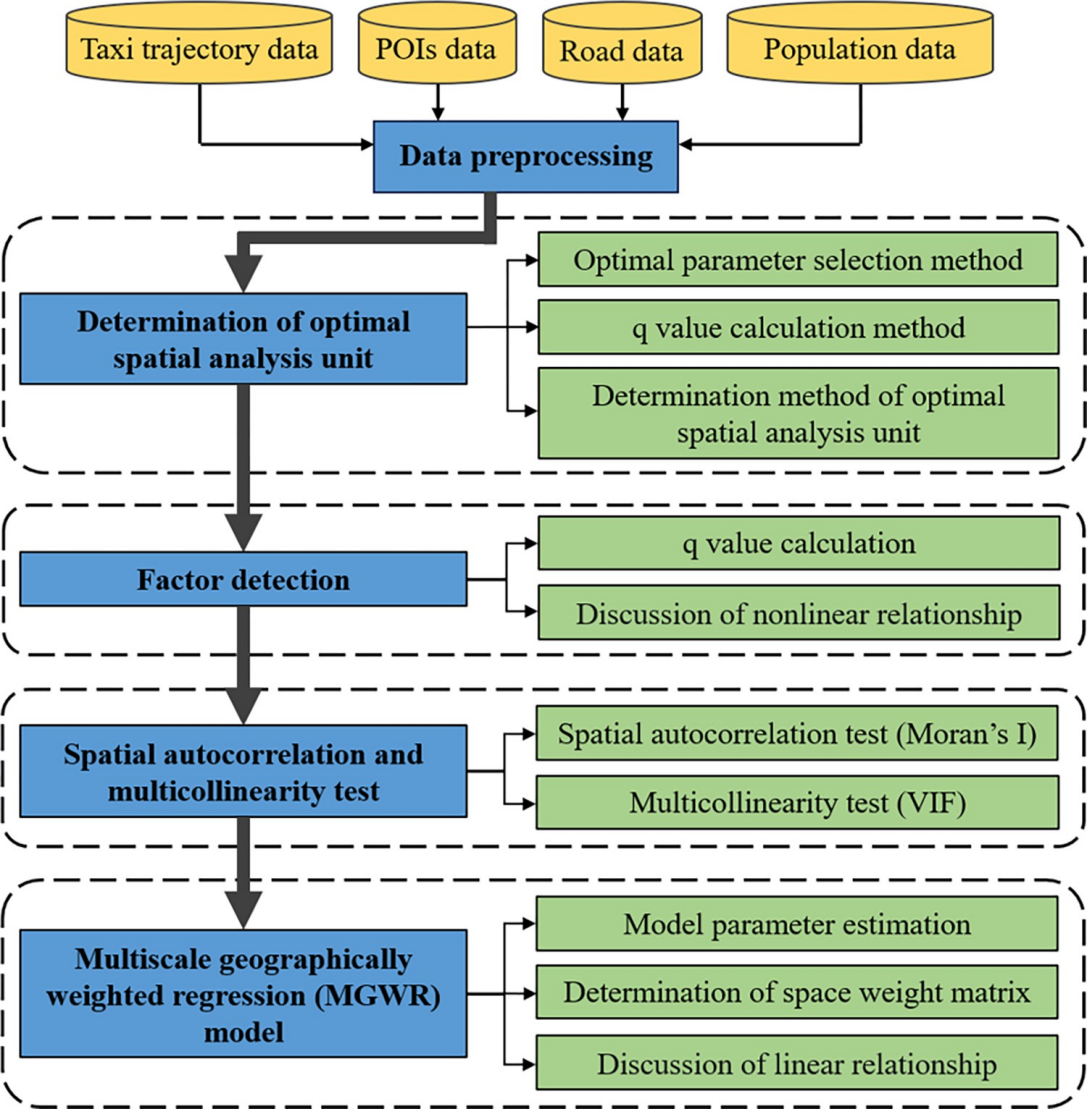
Research framework.

The MAUP and lack of nonlinear and linear relationships were considered to analyze the influence of the built environment on taxi travel demand. First, optimal discretization of the built environment variables under each scale and zoning was performed using the OPGD model. We then calculated the q-value of each built environment variable that affects taxi travel demand as well as the q-value ranking, 90% quantile of the q-values, and their growth rates to select the optimal spatial analysis unit. Furthermore, based on the optimal spatial analysis unit, the nonlinear relationship between the built environment and taxi travel demand was characterized using the factor detection module of the OPGD model. Finally, through spatial autocorrelation and multicollinearity tests of the built environment and taxi travel demand variables, the linear relationship between the built environment and taxi travel demand was characterized using the MGWR model. The overall research framework of the paper is shown in Fig 1.

### 3.1 Determination of the optimal spatial analysis unit

Based on the geographical detector (GD) model proposed by Wang and Xu [49] in 2017, the OPGD model is improved to solve problems such as the lack of an accurate quantitative evaluation of the discretization method and number of classification levels when discretizing continuous variables. The basic principle is the same as that of the GD model, which assumes that the study area is divided into several sub-regions; if the sum of the variances of the sub-regions is less than the total variance of the region, there is spatial heterogeneity. Moreover, if the spatial distribution of two variables tends to be consistent, there is a statistical correlation between the two variables. This model includes four detectors: factor detection, interaction detection, risk detection, and ecological detection. This is a nonlinear statistical method for detecting spatial heterogeneity and revealing its underlying driving forces. The model does not require linear assumptions, it can more reliably explain the relationship between independent and dependent variables, and it is not affected by the multicollinearity of the independent variables. In addition, related studies have shown that this model can solve the MAUP, output model results based on optimal parameters, and detect the impact of individual variables [48, 50]. Therefore, the OPGD model was selected in this paper to determine the optimal spatial analysis unit for the built environment and taxi travel demand. The specific methods are described in the following sections.

#### 3.1.1 Optimal parameter selection method

The numerical independent variable must be discretized into a type-value variable when using the OPGD model. Therefore, the R Programming Language was used in this paper to select the optimal discretization method and classification level number for the built environment variables during discretization. Common discretization methods mainly include equal breaks, natural breaks, quantile breaks, geometric breaks, and standard deviation (SD) breaks. The number of classification levels can be set independently. Finally, by comparing the results of each parameter combination, the parameter combination with the highest q-value for each built environment variable at each scale and zoning was selected as the optimal parameter for geographic detector analysis. The larger the q-value, the better the discretization effect will be [51].

#### 3.1.2 Q-value calculation method

Based on the selection of the optimal parameters, factor detection in the GD model was used to determine the q-value of each built environment variable affecting taxi travel demand at different scales and zones. The calculation is performed as follows:

$$q=1-\frac{1}{n\sigma^{2}}\sum_{h=1}^{L}n_{h}\sigma_{h}^{2}=1-\frac{SSW}{SST}$$ (1)

$$\frac{SSW}{SST}=\sum_{h=1}^{L}n_{h}\sigma_{h}^{2}$$ (2)

$$SST=N\sigma^{2}$$ (3)

where *q* is the explanatory power of the built environment variables, 0<*q*<1. The closer *q* is to 1, the greater the explanatory power of the built environment variable on taxi travel demand will be. In addition, *n* represents the total number of research units, *h* is the stratification of taxi travel demand or built environment variables, *n*_*h*_ is the number of units in layer *h*, *σ*^2^ and $\sigma_{h}^{2}$ represent the overall variance and variance of layer *h*, respectively, *SSW* is the sum of the intra-layer variances, and *SST* is the total variance.

#### 3.1.3 Determination method of the optimal spatial analysis unit

Based on the q-values obtained for each built environment variable affecting taxi travel demand at different scales and zones, the scale effect was evaluated by calculating the ranking of the q-values of the built environment variables at different scales, the 90% quantile of the q-values, and the growth rate. It is generally believed that the more stable the ranking of the q-value [21] or the greater the 90% quantile of the q-values [52], the better the corresponding scale will be. The zoning effect was evaluated by comparing the built environment variable q-values for each zone. Finally, an optimal spatial analysis unit was selected based on the evaluation results.

### 3.2 Factor detection

Based on the optimal spatial analysis unit, the nonlinear relationship between the built environment and taxi travel demand was determined through factor detection in the OPGD model using the q-value to reveal the degree to which the built environment variables explain taxi travel demand. The specific methods have been introduced in optimal parameter selection method and q-value calculation method, so they are not repeated here.

### 3.3 Spatial autocorrelation and multicollinearity tests

#### 3.3.1 Spatial autocorrelation test

Before exploring the linear and spatial heterogeneity relationships between the built environment and taxi travel demand, it is necessary to measure their spatial autocorrelation to determine whether the spatial distribution differences are significant. This paper used global Moran’s I to test the spatial autocorrelation between the built environment and taxi travel demand variables. The calculation is performed as follows:

$$I=\frac{n\sum_{i=1}^{n}\sum_{j=1}^{n}w_{ij}(x_{i}-\bar{x})(x_{j}-\bar{x})}{\sum_{i=1}^{n}\sum_{j=1}^{n}w_{ij}\sum_{i=1}^{n}{\left(x_{i}-\bar{x}\right)}^{2}}$$ (4)

where *I* is the global Moran’s I for taxi travel demand, -1<*I*<1; *n* is the total number of research units; *x*_*i*_ and *x*_*j*_ are the total taxi travel demand of research units *i* and *j*, respectively; $\bar{x}$ is the mean value of the total taxi travel demand of all research units; and *w*_*ij*_ is the space weight matrix. When -1<*I*<0, there is a negative spatial correlation between taxi travel demand in each research unit. When 0<*I*<1, there is a positive spatial correlation between taxi travel demand in each research unit. When *I* = 0, no spatial correlation exists.

#### 3.3.2 Multicollinearity test

When many independent variables are selected, different variables may contain the same information. In this case, there may be multicollinearity between the independent variables. It is necessary to eliminate this multicollinearity before modeling to prevent errors in the model results [53]. Therefore, the variance inflation factor (VIF) test was used to evaluate the multicollinearity of the built environment variables. The calculation formula is:

$$VIF=\frac{1}{1-r_{i}^{2}}$$ (5)

where *VIF* is the variance inflation factor of the built environment variable, *VIF*>1; *r*_*i*_ is the negative correlation coefficient of built environment variable *x*_*i*_ for regression analysis of the remaining built environment variables. When *VIF*<10, no multicollinearity exists between the built environment variables. When *VIF*≥10, it is multicollinearity exists between built environment variables, and appropriate methods should be adopted to adjust it. In this paper, a stepwise regression method was used to screen the built environment variables. This stepwise regression method gradually inputs the built environment variables into the model. If the resulting model is statistically significant, the corresponding variable will be included in the regression model; at the same time, the variables that are not statistically significant are removed from the model. Finally, the built environment variables that meet all conditions are obtained.

### 3.4 Multiscale geographically weighted regression (MGWR) model

The MGWR model was proposed by Fotheringham et al. [54] in 2017. Based on the GWR model, the MGWR model considers the changes in the relationship between independent and dependent variables at different spatial scales (it should be noted that the scale here does not refer to the spatial scale but to the response of the coordinates of different geographical locations to the bandwidth). The advantage of this model is that it overcomes the drawbacks of bandwidth selection, allowing different variables to choose their independent optimal bandwidth to better identify spatial heterogeneity and spatial scales. Furthermore, it improves the goodness-of-fit of the regression model, thus making the regression results faster and more stable, the coefficients more reliable, and the geographical interpretation of the constant terms more meaningful. The basic equation is given as follows:

$$y_{i}=\beta_{i0}(\mu_{i},\nu_{i})+\sum_{k=1}^{m}\beta_{h_{ik}}(\mu_{i},\nu_{i})x_{ik}+\varepsilon_{i}\;i=1,2,3,\ldots ,n$$ (6)

where *y*_*i*_ is the variable for taxi travel demand, *x*_*ik*_ is a built environment variable, *m* is the number of built environment variables, (*μ*_*i*_,*ν*_*i*_) represents the geographical coordinates of the centroid of the *ith* grid, *β*_*i*0_(*μ*_*i*_,*ν*_*i*_) is the intercept of the *ith* grid, $\beta_{h_{ik}}(\mu_{i},\nu_{i})$ is the local regression coefficient of the *kth* built environment variable in the *ith* grid, *h*_*ik*_ is the optimal bandwidth of the *kth* built environment variable in the *ith* grid, and *ε*_*i*_ is a random error in an *ith* grid. Moreover, it follows a normal distribution with a mean of zero and a variance of *δ*^2^.

For the parameter estimation of the MGWR model, this study adopted a back-fitting algorithm and selected the estimated values of the GWR model for the initialization settings. *SOC*_*f*_ was used to determine whether the differences in the parameter estimation converged. To determine the spatial weight matrix, the type of spatial distance was calculated using the Euclidean distance. The type of space weight function was an adaptive bi-square kernel function. The bandwidth selection criteria were the AIC and modified Akaike information criterion (AICc). A detailed description of the relevant parameter estimation and space weight matrix determination, as well as the reasons for selecting the above method, are presented in the Supporting information.

## 4 Study area and data

### 4.1 Study area and division

**Fig 2:**
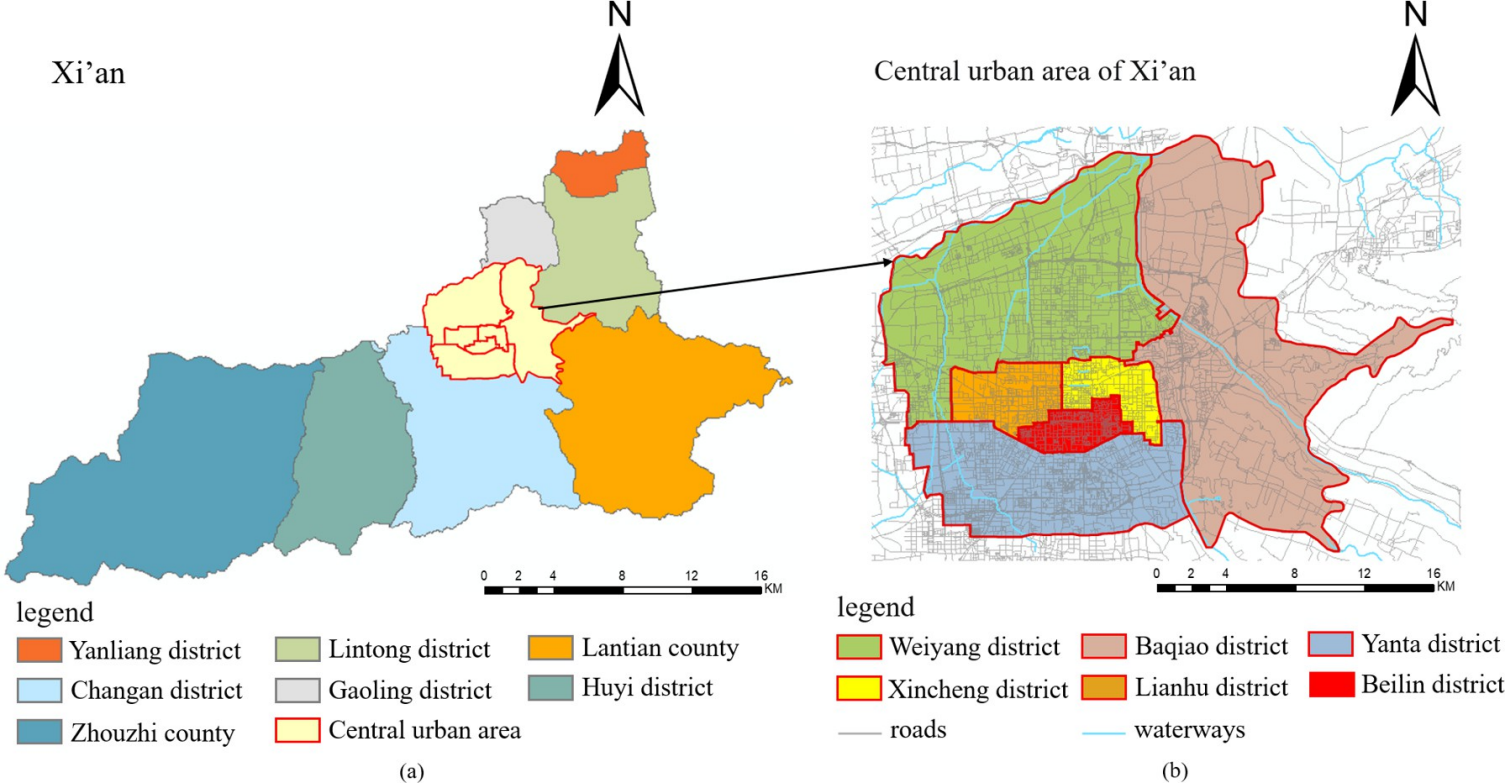
Study area. (a) Study area location; (b) overview of the study area.

As the most populous megacity in northwest China, Xi’an has a longitude range of [107.4, 109.49] and a latitude range of [33.42, 34.45] [55]. At the end of 2022, Xi’an covered an area of 10,108 square kilometers [55] and had a permanent population of 12.9959 million [56]. The city is well-equipped with public transport, and residents’ taxi travel demand is mainly concentrated in the central urban area of Xi’an. Therefore, this study considered the central urban area of Xi’an as the study area (Fig 2(A)), including the districts of Weiyang, Lianhu, Xincheng, Beilin, Yanta, and Baqiao (Fig 2(B)). The study area is approximately 831.87 square kilometers [57].

ArcGIS software was used to divide the study area into grids according to its scope. This classification method was selected because it has strong operability, a wide range of applications, and is suitable for intensive data research. To explore the scale effect of the MAUP, it was necessary to choose the side length of the grid. Considering the scope of the study area and spatial resolution of the multisource data, 100m was chosen as the minimum side length of the grid. A review of the literature on the influence of the built environment on taxi travel demand revealed that most previous studies used 500m or 1000m as the side length of the grid [28, 29, 58, 59]. Therefore, 1000m was selected as the maximum side length, with a side length interval of 100m. To explore the zoning effect of the MAUP, two common grid types were used: regular hexagonal grids and square grids.

**Fig 3:**
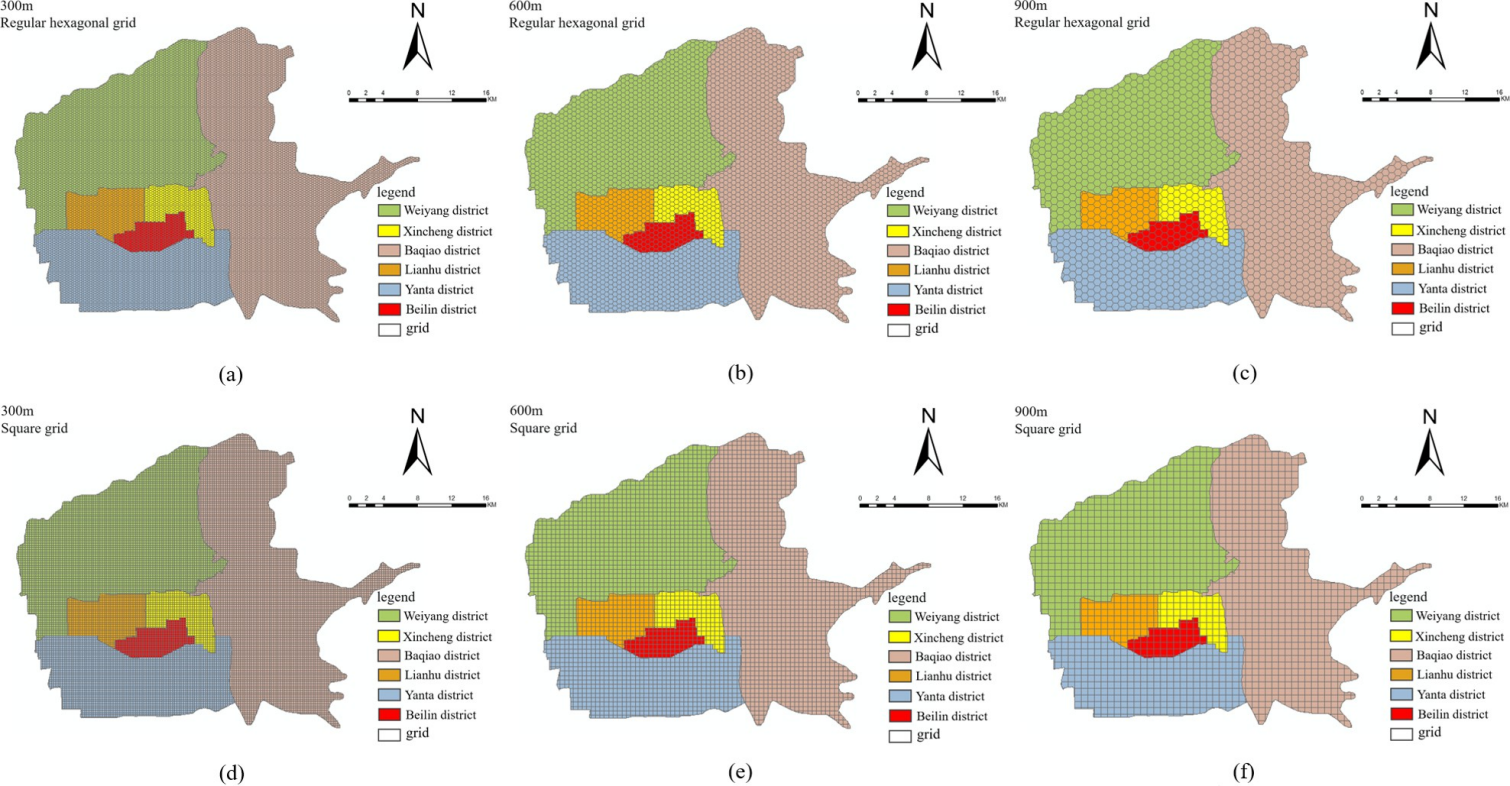
Study area division. (a) 300m regular hexagonal grid; (b) 600m regular hexagonal grid; (c) 900m regular hexagonal grid; (d) 300m square grid; (e) 600m square grid; (f) 900m square grid.

The specific operation method created regular hexagonal grids with ten scales and side lengths ranging from 100m to 1000m (with an interval of 100m). The areas of the regular hexagonal grids were then fixed at each scale, and corresponding square grids were generated (a total of 10). Ultimately, 20 types of research units were generated. Here, only the 300m, 600m, and 900m scales are taken as examples, and the study area is divided into regular hexagonal and square grids, as shown in Fig 3.

### 4.2 Data sources and processing

#### 4.2.1 Taxi travel demand data

The taxi travel demand data used in this paper were obtained from taxi GPS trajectory data provided by the taxi management office of Xi’an city. The selected time range was 30 days, from March 1, 2019 to March 30, 2019. Owing to the extensive period of the selected data, the data volume was also large. Moreover, there were no special holidays during this period; therefore, the data was assumed to be objective and representative. The original trajectory data included the Vehicle ID, Intime, Time, Longitude, and Latitude. An example of the taxi trajectory data structure is presented in Table 1.

**Table 1:** An instance of taxi trajectory data structure.

Name	Sample	Type	Meaning
Vehicle ID	AT3607	Char	The issuance of vehicle license plates
Intime	2019/3/1 07:15:35	Char	Storage time
Time	2019/3/1 07:15:34	Char	GPS_time
Longitude	108.784927	Float	Location information
Latitude	34.746352	Float	Location information
Height	0	Short	elevation
Speed	35	Short	level speed
Direction	90	Short	Front direction
EFF	1	Char	Vehicle state bit; 1 means valid, 0 means invalid
State	5	Char	Vehicle state; 4 means empty, 5 means someone in the vehicle

**Fig 4:**
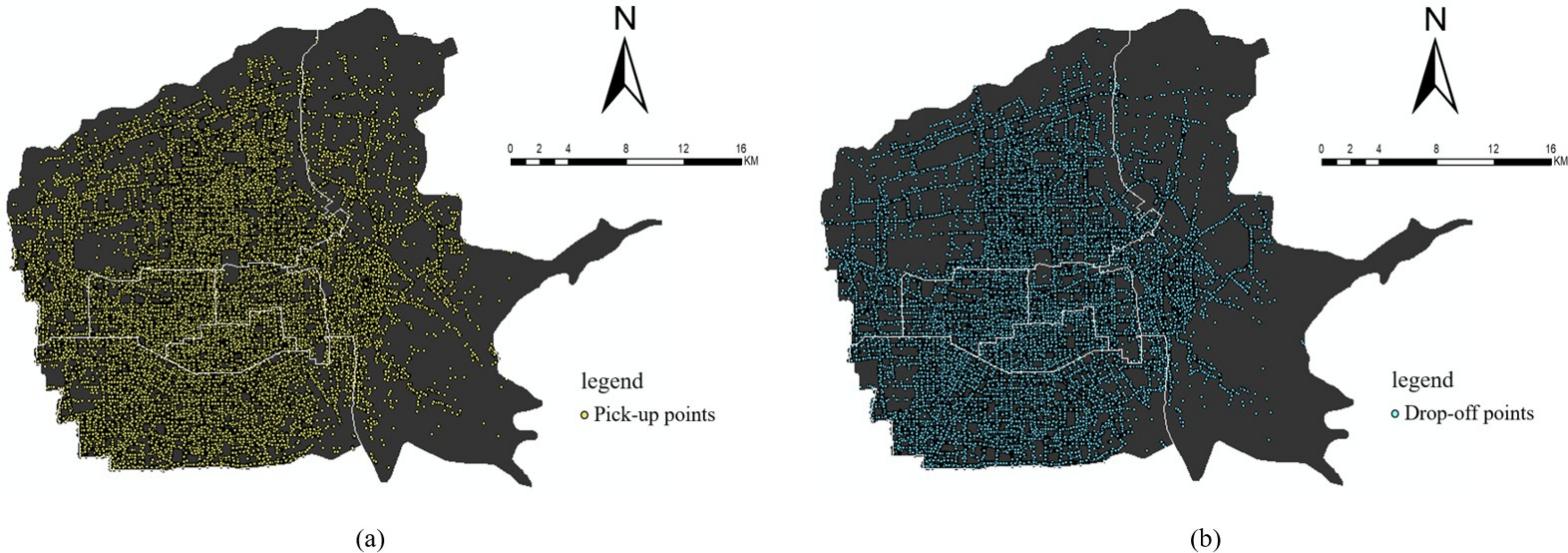
Density of taxi pick-up and drop-off points. (a) Pick-up points; (b) drop-off points.

The total original trajectory data obtained in this paper was approximately 11.6 million. Python software was then used to remove the driving records with outliers and those occurring outside the central urban area of Xi’an. Data corresponding to pick-up points (the points at which the vehicle state changed from “4” to “5”) and drop-off points (the points at which the vehicle state changed from “5” to “4”) were then extracted as the taxi travel demand data for the central urban area of Xi’an. Finally, a total of 10.3 million effective taxi trajectory data were obtained. In other words, 88.79% of the valid data was obtained. It was considered that displaying all of the taxi pick-up and drop-off points for 30 days was prone to data being tightly distributed and unattractive. Therefore, the simple random sampling method was adopted to display the density distribution based on 5% of the taxi pick-up and drop-off points in the central urban area of Xi’an after cleaning and treatment (Fig 4).

#### 4.2.2 Built environment data

Based on the “5D” dimensions of built environment characteristics, this paper selected built environment variables from the five dimensions of density, design, diversity, destination accessibility, and distance to transit. There were three primary sources of data. First, population data were obtained from the Seventh National Population Census of Xi’an City, China (http://tjj.xa.gov.cn). Data were collected at the street scale. Then, it was processed on a grid scale as the built environment variable for the density dimension according to the research scope. Second, POI data representing various facilities were obtained from the 2019 Xi’an data on the Amap open platform (https://lbs.amap.com/). Reptiles were collected to obtain 13 first-level POI categories, including shopping service POI, catering service POI, accommodation services POI, etc., which were used to quantify the built environment variables of the density dimension. Based on the POI data, the mixed degree of urban functions was calculated as the built environment variable for the diversity dimension. In addition, bus stop POI and subway station POI were extracted to quantify the built environment variables in the distance-to-transit dimension. In addition, road data was obtained from Xi’an in 2019 on OpenStreetMap (the OSM data download address in China is https://download.geofabrik.de/). According to the study area and classification of the road grade, roads were quantified into four levels: primary roads, secondary roads, tertiary roads, and urban express roads, which were used to represent the built environment variables of the design dimension. Finally, the Euclidean distance from each grid centroid to the city center was used to quantify the built environment variables in the destination accessibility dimension. The main variables for each dimension and their calculation methods are shown in Table 2.

**Table 2:** The “5D” dimension of built environment variables.

“5D” dimension	Variables title	Description	Calculation method
Density	P_d	Population density	Intersecting street-scale population data with the grid. Calculate the proportion of street area corresponding to each grid after intersection. Then multiply the population data with the corresponding street area ratio of the grid as the population data at the grid scale. Population density is the ratio of population to total area within each scale and zoning
	Cs_d	Catering service POI density	The ratio of the number of catering services to the total area within each scale and zoning
	Ta_d	Tourist attraction POI density	The ratio of the number of tourist attractions to the total area within each scale and zoning
	Pa_d	Public facility POI density	The ratio of the number of public facilities to the total area within each scale and zoning
	Ss_d	Shopping service POI density	The ratio of the number of shopping services to the total area within each scale and zoning
	Tf_d	Transportation facility POI density	The ratio of the number of transportation facilities to the total area within each scale and zoning
	Et_d	Education and training POI density	The ratio of the amount of education and training to the total area within each scale and zoning
	Fis_d	Financial and insurance service POI density	The ratio of the number of financial and insurance services to the total area within each scale and zoning
	Cr_d	Commercial residence POI density	The ratio of the number of commercial residences to the total area within each scale and zoning
	Ls_d	Life service POI density	The ratio of the number of life services to the total area within each scale and zoning
	Sls_d	Sports and leisure service POI density	The ratio of the number of sports and leisure services to the total area within each scale and zoning
	Ms_d	Medical service POI density	The ratio of the number of medical services to the total area within each scale and zoning
	Gaso_d	Government agency and social organization POI density	The ratio of the number of government agencies and social organizations to the total area within each scale and zoning
	As_d	Accommodation services POI density	The ratio of the number of accommodation services to the total area within each scale and zoning
Diversity	Mduf	Mixed degree of urban functions	The spatial information entropy of mixed degree of urban functions: $H_{s_{ij}}=-\sum_{i}^{m}\sum_{j}^{n}p_{ij}\;\log\;p_{ij}$ Where $H_{s_{ij}}$ is the spatial information entropy of mixed degree of urban functions. *p*_*ij*_ is in the grid of row *i* and column *j*, the ratio of the number of certain POI types in the total number. $\sum_{i}\sum_{j}p_{ij}=1$.
Design	Prn_d	Primary road network density	The ratio of the length of primary roads to the total area within each scale and zoning
	Srn_d	Secondary road network density	The ratio of the length of secondary roads to the total area within each scale and zoning
	Trn_d	Tertiary road network density	The ratio of the length of tertiary roads to the total area within each scale and zoning
	Uern_d	Urban express road network density	The ratio of the length of urban express roads to the total area within each scale and zoning
Destination accessibility	Dcc	Distance to the city center	The straight-line distance from the centroid to the city center within each scale and zoning
Distance to transit	Bs_d	Bus stop POI density	The ratio of the number of bus stops to the total area within each scale and zoning
	Ss_d	Subway station POI density	The ratio of the number of subway stations to the total area within each scale and zoning

## 5 Results and discussion

### 5.1 Determination result of the optimal spatial analysis unit

According to the above description of the OPGD model, this paper discretized the built environment variables at different scales and zones using five methods: equal breaks, natural breaks, quantile breaks, geometric breaks, and SD breaks, and the classification levels were divided into 3–10 categories. The optimal discretization of built environment variables was obtained using R Programming Language (see Supporting information for the results of the optimal discretization of the built environment variables).

**Fig 5:**
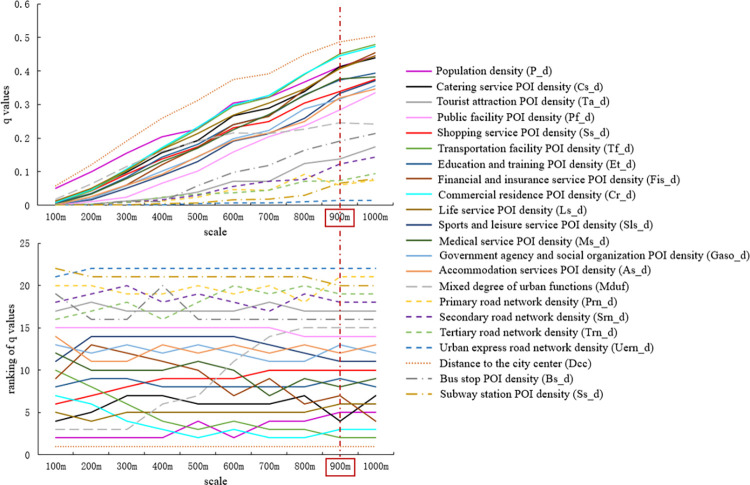
q-values of built environment variables at different scales and their ranking (regular hexagonal grid).

**Fig 6:**
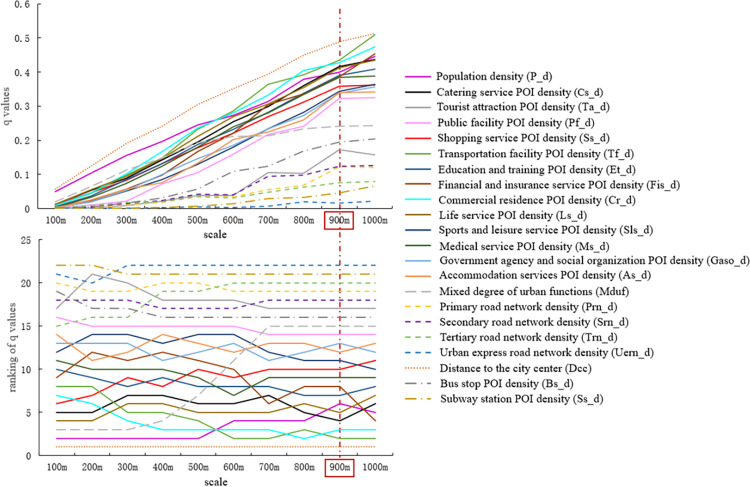
q-values of built environment variables at different scales and their ranking (square grid).

Furthermore, the q-values of the built environment variables were calculated according to the optimal discretization results for the built environment variables at different scales and zones. The q-values of each built environment variable were then ranked at different scales and zones to represent the influence stability of each variable (the results are shown in Figs 5 and 6). A smaller fluctuation in the ranking of the q-values for each variable with the change in scale indicates that the influence is more stable, and the scale of the corresponding spatial analysis unit is more appropriate.

As shown in Figs 5 and 6, regardless of whether the regular hexagonal grid or square grid, the influence of most built environment variables increased with an increase in scale. This phenomenon was particularly evident when the scale was less than 900m. In addition, regardless of the regular hexagonal or square grid used, the influence of the ranking of built environment variables differed at different scales. This indicates that different scales had different effects on taxi travel demand. For instance, the rankings of the public facility POI density (Pf_d), urban express road network density (Uern_d), distance to the city center (Dcc), and subway station POI density (Ss_d) were relatively stable across all scales, indicating that they had low scale sensitivity for the influence of taxi travel demand. However, the q-values of the other built environment variables and their rankings were sensitive to scale. Urban planners and managers should thus pay attention to the spatial scale when considering the influence of these built environment variables. When the scale was greater than 900m, the fluctuations in most of the built environment variables decreased. Based on this analysis, the 900m scale was selected as the initial candidate for the optimal spatial unit for the regular hexagonal and square grids.

**Fig 7:**
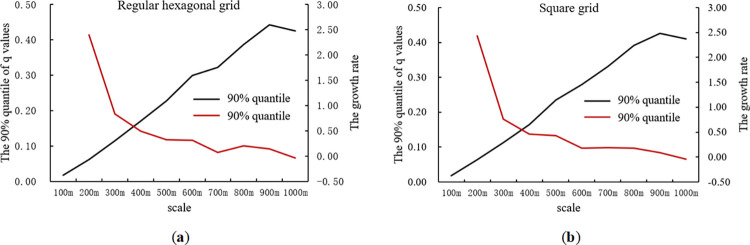
The 90% quantile of the q-values of built environment variables and their growth rates at each scale. (a) Regular hexagonal grid; (b) square grid.

Furthermore, the 90% quantiles of the q-values and their growth rates were calculated for the built environment variables at different scales and zones. The results are shown in Fig 7.

Fig 7 shows that for both the regular hexagonal grid and the square grid, the 90% quantile of the q-values of the built environment variables gradually increased with increasing scale. However, when the scale exceeded 900m, the 90% quantile of q-values decreases. That is, when the scale was 900m, the 90% quantile of the q-values of the built environment variables reached its maximum value. The growth rate of the 90% quantile of the q-values showed a trend of decreasing, followed by a slight increase and then a further decrease. The growth rate of the 90% quantile of the q-values of built environment variables was relatively large before the 900m scale. When the scale exceeded 900m, the growth rate decreased. Therefore, it is possible to select the 900m scale as the optimal spatial analysis unit for regular hexagonal and square grids. Finally, the q-values of each built environment variable corresponding to the regular hexagonal grid and square grid at the 900m scale were compared. The results are presented in Table 3.

**Table 3:** q-values of each built environment variable at 900m scale.

Built environment variable	q-value (regular hexagonal grid)	q-value (square grid)
Population density (P_d)	0.4124	0.4000
Catering service POI density (Cs_d)	0.4162	0.4128
Tourist attraction POI density (Ta_d)	0.1719	0.1381
Public facility POI density (Pf_d)	0.3230	0.2839
Shopping service POI density (Ss_d)	0.3576	0.3384
Transportation facility POI density (Tf_d)	0.4503	0.4345
Education and training POI density (Et_d)	0.3733	0.3896
Financial and insurance service POI density (Fis_d)	0.4061	0.3871
Commercial residence POI density (Cr_d)	0.4454	0.4267
Life service POI density (Ls_d)	0.4116	0.4072
Sports and leisure service POI density (Sls_d)	0.3425	0.3322
Medical service POI density (Ms_d)	0.3763	0.3851
Government agency and social organization POI density (Gaso_d)	0.3158	0.3386
Accommodation services POI density (As_d)	0.3389	0.3198
Mixed degree of urban functions (Mduf)	0.2463	0.2410
Primary road network density (Prn_d)	0.1214	0.0608
Secondary road network density (Srn_d)	0.1247	0.1229
Tertiary road network density (Trn_d)	0.0747	0.0734
Urban express road network density (Uern_d)	0.0138	0.0160
Distance to the city center (Dcc)	0.4882	0.4869
Bus stop POI density (Bs_d)	0.1940	0.1908
Subway station POI density (Ss_d)	0.0651	0.0465

As indicated in Table 3, at the 900m scale, the q-values of each built environment variable based on the regular hexagonal grid were generally larger than those based on the square grid. This indicates that the model performance of the regular hexagonal grid division scheme was slightly better than that of the square grid division scheme. Correlation analysis has also shown that using a regular hexagonal grid as the spatial analysis unit can reduce the visual field deviation compared with a square grid [60].

In summary, based on the scope of the study area, the optimal spatial analysis unit was selected as a regular hexagonal grid with a side length of 900m.

### 5.2 Result of factor detection

This paper analyzed the influence of the built environment on the total demand for taxi pick-up and drop-off on weekdays and weekends and obtained results for the difference between weekdays and weekends. Prior to the analysis, an optimal discrete selection of built environment variables corresponding to taxi travel demand variables was performed. The results are summarized in Table 4.

**Table 4:** The optimal discretization results of built environment variables on weekdays and weekends.

“5D” dimension	Built environment variable	Weekdays		Weekends	
		Discretization method	Classification number	Discretization method	Classification number
Density	P_d	natural	10	natural	10
	Cs_d	quantile	7	quantile	7
	Ta_d	sd	7	sd	7
	Pa_d	sd	10	sd	10
	Ss_d	quantile	10	quantile	10
	Tf_d	natural	9	natural	9
	Et_d	natural	9	quantile	10
	Fis_d	sd	10	sd	10
	Cr_d	natural	10	natural	10
	Ls_d	quantile	10	quantile	10
	Sls_d	sd	10	natural	10
	Ms_d	quantile	9	quantile	9
	Gaso_d	natural	10	natural	10
	As_d	quantile	10	quantile	10
Diversity	Mduf	quantile	9	quantile	9
Design	Prn_d	quantile	8	natural	10
	Srn_d	sd	10	sd	10
	Trn_d	sd	10	sd	10
	Uern_d	natural	10	natural	10
Destination accessibility	Dcc	sd	9	geometric	10
Distance to transit	Bs_d	sd	5	sd	5
	Ss_d	quantile	3	equal	3

Furthermore, according to the optimal discretization results for the built environment variables, the factor detector was used to explore the independent influence of the built environment variables on the total demand for taxi pick-up and drop-off on weekdays and weekends. The results are provided in Table 5.

**Table 5:** The independent effect of built environment variables on the total demand for taxi pick-up and drop-off.

“5D” dimension	Built environment variable	Weekdays		Weekends	
		q value	P-value	q value	P-value
Density	P_d	0.4158	0.0000	0.4111	0.0000
	Cs_d	0.4137	0.0000	0.4054	0.0000
	Ta_d	0.1349	0.0000	0.1447	0.0000
	Pa_d	0.2831	0.0000	0.2827	0.0000
	Ss_d	0.3387	0.0000	0.3334	0.0000
	Tf_d	0.4451	0.0000	0.4365	0.0000
	Et_d	0.3791	0.0000	0.3686	0.0000
	Fis_d	0.4081	0.0000	0.3963	0.0000
	Cr_d	0.4460	0.0000	0.4318	0.0000
	Ls_d	0.4082	0.0000	0.3996	0.0000
	Sls_d	0.3314	0.0000	0.3221	0.0000
	Ms_d	0.3758	0.0000	0.3729	0.0000
	Gaso_d	0.3204	0.0000	0.3260	0.0000
	As_d	0.3201	0.0000	0.3152	0.0000
Diversity	Mduf	0.2485	0.0000	0.2377	0.0000
Design	Prn_d	0.0654	0.0000	0.0614	0.0000
	Srn_d	0.1241	0.0000	0.1183	0.0000
	Trn_d	0.0743	0.0000	0.0704	0.0000
	Uern_d	0.0139	0.0525	0.0128	0.0763
Destination accessibility	Dcc	0.4857	0.0000	0.4858	0.0000
Distance to transit	Bs_d	0.1927	0.0000	0.1834	0.0000
	Ss_d	0.0646	0.0000	0.0654	0.0000

Table 5 indicates that on both weekdays and weekends, only the Uern_d variable in the design dimension did not pass the significance test, i.e., the P-value was more significant than 5%. All of the other built environment variables passed the significance test and thus had statistical significance. In general, the built environment variables were ranked as destination accessibility > density > diversity > distance to transit > design according to the “5D” dimensions and influence. Specifically, the top variables of the built environment in terms of influence were Dcc, Commercial residence POI density (Cr_d), Transportation facility POI density (Tf_d), Population density (P_d), Catering service POI density (Cs_d), Life service POI density (Ls_d), and Financial and insurance service POI density (Fis_d). At the bottom of the rankings were Uern_d and Ss_d. Moreover, the influence of built environment variables was generally higher on weekdays than on weekends. Only the Tourist attraction POI density (Ta_d), Government agency and social organization POI density (Gaso_d), Dcc, and Ss_d had a lower influence on weekdays than on weekends. In particular, the influence of Ta_d on weekdays was lower than that on weekends compared with the other variables. In contrast, the influence of the Public facility POI density (Pf_d) and Dcc was similar on weekdays and weekends.

In summary, the influence of built environment variables on the total demand for taxi pick-up and drop-off on weekdays and weekends reveals the following: (1) In the density dimension, the influence of Ta_d on weekends was higher than that on weekdays, which confirms the weekend effect of tourism. (2) In the diversity dimension, the influence of the Mixed degree of urban functions (Mduf) was moderate in the overall built environment variable. (3) In the design dimension, the influence of the Secondary road network density (Srn_d) was stronger than those of the Primary road network density (Prn_d), Tertiary road network density (Trn_d), and Uern_d. This indicates that Srn_d in road design is the main reason for the difference in the distribution of the total demand for taxi pick-up and drop-off. In addition, the influence of Uern_d was the lowest among all of the built environment variables. (4) In the destination accessibility dimension, Dcc had the greatest influence on the overall built environment variables. This shows that different urban locations can lead to large differences in the total demand for taxi pick-up and drop-off. (5) In the distance to transit dimension, the influence of the Bus stop POI density (Bs_d) was much higher than that of Subway station POI density (Ss_d). This indicates that the Bs_d distribution is the most important transportation factor affecting the total demand for taxi pick-up and drop-off.

### 5.3 Results of spatial autocorrelation and multicollinearity test

#### 5.3.1 Results of the spatial autocorrelation test

Moran’s I was used to measure the global spatial autocorrelation of all taxi travel demand and built environment variables, as listed in Table 6.

**Table 6:** The global Moran’s I of taxi travel demand variable and built environment variable.

Variable		Moran’s I	Expected index	Variance	Z-score	P-value
Taxi travel demand variable	Total demand for taxi pick-up and drop-off on weekdays	0.6704	-0.0006	0.0002	46.1017	0.0000
	Total demand for taxi pick-up and drop-off on weekends	0.6613	-0.0006	0.0002	45.5086	0.0000
Built environment variable	P_d	0.9362	-0.0006	0.0002	64.2191	0.0000
	Cs_d	0.5926	-0.0006	0.0002	40.8822	0.0000
	Ta_d	0.3290	-0.0006	0.0003	25.2202	0.0000
	Pa_d	0.5503	-0.0006	0.0002	38.2850	0.0000
	Ss_d	0.5021	-0.0006	0.0002	34.5760	0.0000
	Tf_d	0.7470	-0.0006	0.0002	51.3145	0.0000
	Et_d	0.6202	-0.0006	0.0002	43.0679	0.0000
	Fis_d	0.5251	-0.0006	0.0002	36.7713	0.0000
	Cr_d	0.7727	-0.0006	0.0002	53.0827	0.0000
	Ls_d	0.6059	-0.0006	0.0002	41.7116	0.0000
	Sls_d	0.5773	-0.0006	0.0002	39.9151	0.0000
	Ms_d	0.5954	-0.0006	0.0002	41.0438	0.0000
	Gaso_d	0.6025	-0.0006	0.0002	41.6919	0.0000
	As_d	0.395856	-0.0006	0.0002	27.8000	0.0000
	Mduf	0.6327	-0.0006	0.0002	43.3647	0.0000
	Prn_d	0.1949	-0.0006	0.0002	14.5026	0.0000
	Srn_d	0.3985	-0.0006	0.0002	27.3793	0.0000
	Trn_d	0.2464	-0.0006	0.0002	19.3892	0.0000
	Uern_d	0.3191	-0.0006	0.0002	22.1648	0.0000
	Dcc	0.9943	-0.0006	0.0002	68.1434	0.0000
	Bs_d	0.3563	-0.0006	0.0002	24.4971	0.0000
	Ss_d	0.0649	-0.0006	0.0002	4.5056	0.0000

As presented in Table 6, the global Moran’s I of all taxi travel demand variables and built environment variables were greater than zero, and the P-value was less than 0.05. This indicates a significant positive spatial correlation at the 95% confidence level. In addition, the Z-scores were all greater than 1.96, indicating that all variables had spatial agglomeration characteristics. These are consistent with the preconditions for the MGWR.

#### 5.3.2 Results of the multicollinearity test

The VIF was used to conduct a multicollinearity test on the built environment variables; the results are provided in Table 7.

**Table 7:** Results of multicollinearity test of built environment variables on weekdays and weekends.

“5D” dimension	Built environment variable	VIF (weekdays)	VIF (weekends)
Density	P_d	2.7433	2.7433
	Cs_d	10.5986	10.5986
	Ta_d	1.2091	1.2091
	Pa_d	2.5405	2.5405
	Ss_d	2.8073	2.8073
	Tf_d	6.7096	6.7096
	Et_d	2.3768	2.3768
	Fis_d	3.6789	3.6789
	Cr_d	5.2999	5.2999
	Ls_d	9.3920	9.3920
	Sls_d	4.7389	4.7389
	Ms_d	3.1731	3.1731
	Gaso_d	2.4264	2.4264
	As_d	2.7100	2.7100
Diversity	Mduf	2.2083	2.2083
Design	Prn_d	1.1283	1.1283
	Srn_d	1.1719	1.1719
	Trn_d	1.0328	1.0328
	Uern_d	——	——
Destination accessibility	Dcc	3.0820	3.0820
Distance to transit	Bs_d	1.7548	1.7548
	Ss_d	1.2106	1.2106

Table 7 reveals that only the VIF of Cs_d was greater than 10. This indicates that there is multicollinearity among the built environment variables. Furthermore, we used SPSS software to screen the important variables with a significant influence using a stepwise regression method. The variable screening results are shown in Table 8.

**Table 8:** Stepwise regression screening results for significant variables on weekdays and weekends.

Built environment variable	Weekdays				Weekends			
	Coef.	t	P-value	VIF	Coef.	t	P-value	VIF
Intercept	0	5.52	0.000***	——	0	5.316	0.000***	——
P_d	0.212	8.165	0.000***	2.368	0.21	7.986	0.000***	2.368
Cs_d	0.082	2.696	0.007***	3.253	0.099	3.227	0.001***	3.253
Tf_d	0.247	8.609	0.000***	2.881	0.223	7.709	0.000***	2.881
Ms_d	0.094	3.421	0.001***	2.648	0.103	5.654	0.000***	1.134
Srn_d	0.107	5.977	0.000***	1.134	0.102	3.679	0.000***	2.648
Dcc	-0.164	-6.652	0.000***	2.131	-0.161	-6.458	0.000***	2.131

Note

***, ** and * represent the significance level of 1%, 5% and 10% respectively.

The results in Table 8 demonstrate that after stepwise regression, the selected built environment variables did not have multicollinearity and were all significant. Thus, these variables could be used as the input variables for the MGWR model.

### 5.4 Results of spatial heterogeneity analysis

#### 5.4.1 Results of model evaluation

We established MGWR models based on the selected built environment and taxi travel demand variables; GWR Models were simultaneously constructed as controls. A comparison of the results is presented in Table 9.

**Table 9:** Comparison results of the GWR and the MGWR models on weekdays and weekends.

	Model	*R* ^2^	$\bar{R^{2}}$	*AIC*	*AICc*	*RSS*
Weekdays	GWR	0.6367	0.6186	3159.2876	3194.0361	539.7600
	MGWR	0.7330	0.7020	2933.9270	2974.6880	454.5340
Weekends	GWR	0.6289	0.6104	3176.2938	3228.1504	557.4190
	MGWR	0.7260	0.6950	2975.8360	3016.4890	465.9920

Table 9 reveals that the *R*^2^ values and $\bar{R^{2}}$ values of the MGWR model were higher than those of the GWR model for both weekdays and weekends. Moreover, the values of *AIC*, *AICc*, and *RSS* were also lower than those of the GWR model. Thus, it can be concluded that the MGWR model has a better fitting effect and explanatory power than the GWR model. In addition, the difference between the *AICc* values in the MGWR model and the GWR model was greater than three, indicating that the MGWR model is superior to the GWR model. A bandwidth comparison between the GWR and MGWR models is presented in Table 10.

**Table 10:** Comparison of bandwidth between the GWR and MGWR models on weekdays and weekdays.

Built environment variable	weekdays		weekends	
	GWR bandwidth	MGWR bandwidth	GWR bandwidth	MGWR bandwidth
Intercept	1003	51	1016	51
P_d	1003	1700	1016	1700
Cs_d	1003	1661	1016	1661
Tf_d	1003	1309	1016	1700
Ms_d	1003	838	1016	838
Srn_d	1003	51	1016	51
Dcc	1003	896	1016	1179

Table 10 indicates that the GWR model and MGWR model have vastly different results in terms of bandwidth. The bandwidths of each variable in the GWR model are fixed, whereas those in the MGWR model vary significantly. In other words, the action scales of different built environment variables are quite different. The P_d, Srn_d, Medical service POI density (Ms_d), and Cs_d variables have the same action scales of 1700, 51, 838, and 1661, respectively, accounting for 99.94%, 3.00%, 49.27%, and 97.65% of the total samples, respectively, for both weekdays and weekends. The results show that P_d, Ms_d, and Cs_d have small spatial heterogeneity. In contrast, Srn_d has large spatial heterogeneity, and the effects of these variables are the same on weekdays and weekends. In addition, the action scales of Tf_d on weekdays and weekends are 1309 and 1700, respectively, accounting for 76.95% and 99.94% of the total samples, respectively. The results show little spatial heterogeneity and a certain difference between weekdays and weekends. The action scales of Dcc on weekdays and weekends are 896 and 1179, respectively, accounting for 52.67% and 69.31% of the total samples, respectively, similar to the results for Tf_d.

In summary, the MGWR model was superior to the GWR model in terms of fit. Moreover, the spatial scale of the relationship between different built environment variables was considered. The MGWR model could fit the taxi travel demand well and further reveal the spatial differences in the influence of the built environment on taxi travel demand. Therefore, the MGWR model was used to elaborate the spatial heterogeneity of the degree of impact of the built environment on taxi travel demand.

#### 5.4.2 Spatial heterogeneity analysis of regression coefficients

**Fig 8:**
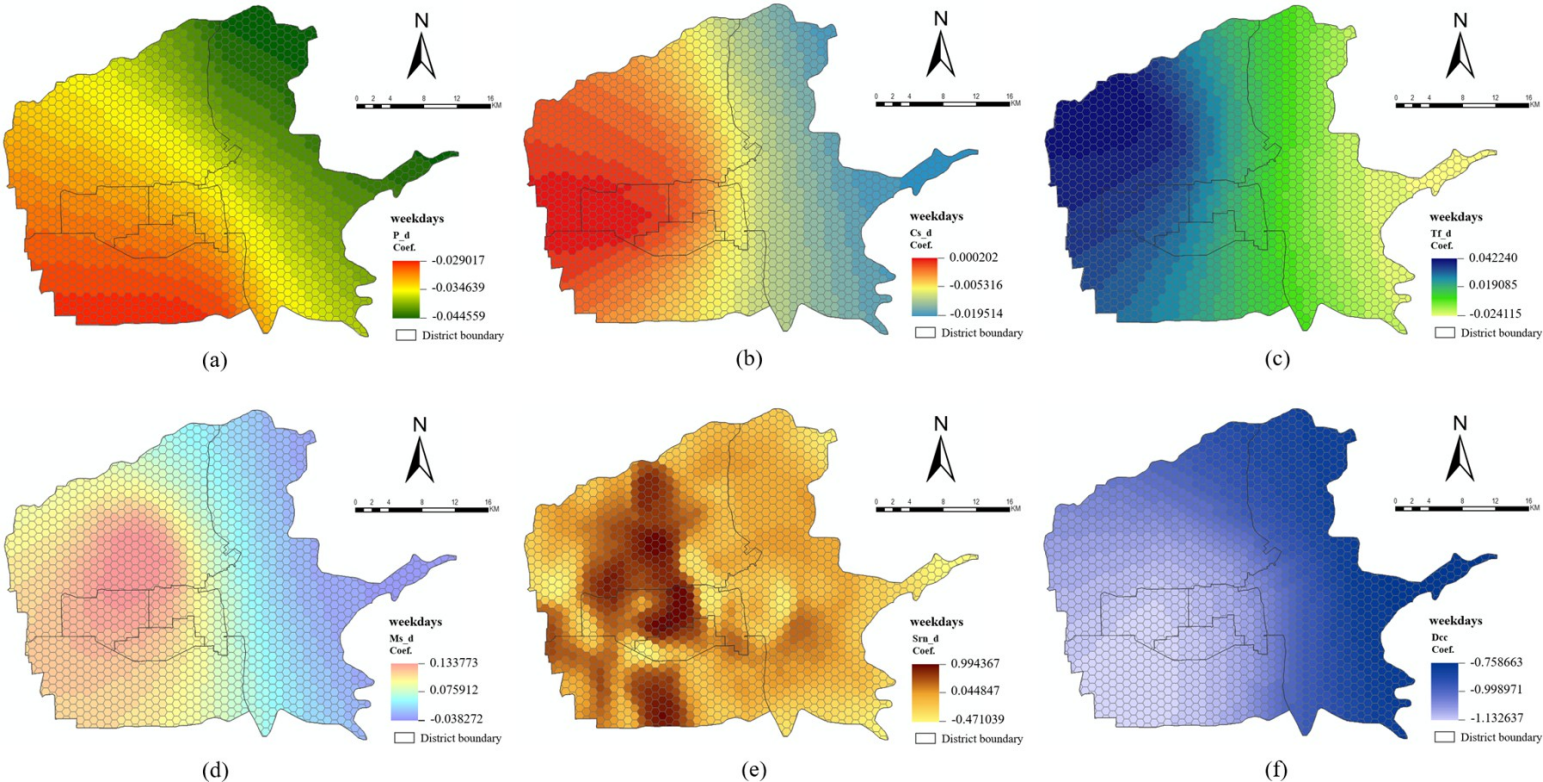
Spatial distribution of local regression coefficients of built environment variables on weekdays. (a) P_d; (b) Cs_d; (c) Tf_d; (d) Ms_d; (e) Srn_d; (f) Dcc.

**Fig 9:**
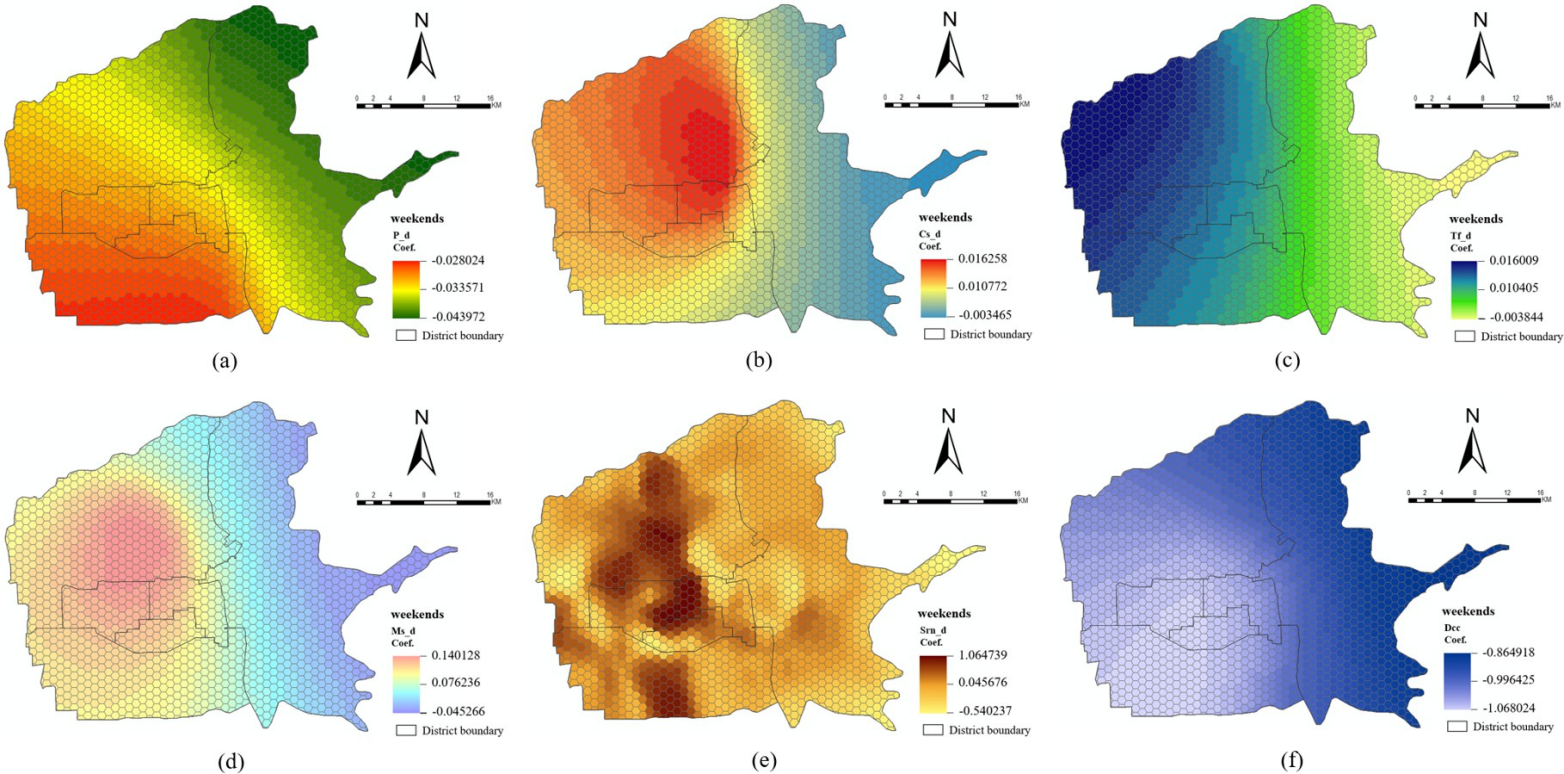
Spatial distribution of local regression coefficients of built environment variables on weekends. (a) P_d; (b) Cs_d; (c) Tf_d; (d) Ms_d; (e) Srn_d; (f) Dcc.

The local regression coefficients were visualized to demonstrate the influence of built environment variables on taxi travel demand in different spatial positions on weekdays and weekends (Figs 8 and 9). Positive and negative symbols indicate a positive or negative influence of each built environment variable on taxi travel demand. The absolute values indicate the degree of influence.

*Density dimension*. The regression coefficients for P_d on weekdays and weekends are both negative (Figs 8(A) and 9(A)), indicating that P_d has a negative inhibitory effect on the total demand for taxi pick-up and drop-off. The overall negative inhibitory effect gradually increases from the southwest to the northeast. The areas where P_d has a large negative inhibitory effect on the total demand for taxi pick-up and drop-off on weekdays and weekends are located in the northeast and east of the Baqiao District. Because of the low level of economic development and resident income in these areas, and because most of the area is under development and construction, residents are more willing to choose buses and subways as their main means of transportation. Therefore, compared with the less strongly negative inhibitory effect in the southeast of Yanta District, which is relatively developed and has a high resident income, P_d has a greater influence on taxi travel demand.

The regression coefficients of Cs_d on weekdays and weekends are both positive and negative (Figs 8(B) and 9(B)). This indicates that in some regions, Cs_d has a positive promoting effect on the total demand for taxi pick-up and drop-off. In contrast, in some regions, Cs_d has a negative inhibitory effect on the total demand for taxi pick-up and drop-off. The areas where Cs_d has a significant negative inhibitory effect on the total demand for taxi pick-up and drop-off on weekdays and weekends are all located in the east of the Baqiao District. Cs_d on weekdays has a significant positive influence on the total demand for taxi pick-up and drop-off, particularly in most areas of the Lianhu District and at the junction of the Lianhu District, Xincheng District, and Beilin District. The areas with a significant positive promotion effect on weekends include most areas of the Xincheng District and the southeast of the Weiyang District. This change indicates a difference between weekdays and weekends in areas where Cs_d has a greater positive influence on the total demand for taxi pick-up and drop-off.

The regression coefficients of Tf_d on weekdays and weekends are both positive and negative (Figs 8(C) and 9(C)), and the overall spatial distribution of the regression coefficients is not significantly different. The overall trend is from east to west; in this direction, the negative inhibitory effect gradually weakens and the positive promoting effect gradually increases. The negative inhibitory effect of Tf_d on the total demand for taxi pick-up and drop-off on weekdays and weekends is strongest in the east of the Baqiao District. The positive promoting effect is strongest in the northwest of the Weiyang District. Closer to the central urban area, from south to north, both the negative inhibitory and positive promotion effects are weakened. Because many transportation facilities such as subway stations and bus stops are available for residents to travel along this section, the increase or decrease in transportation facilities has less influence on the total demand for taxi pick-up and drop-off compared with other areas.

The regression coefficients of Ms_d on weekdays and weekends are both positive and negative (Figs 8(D) and 9(D)), and the overall spatial distribution of the regression coefficients is not significantly different. The areas with a significant negative inhibitory effect of Ms_d on the total demand for taxi pick-up and drop-off on weekdays and weekends are located in the east, northeast, and southeast of the Baqiao District. Residents in these areas have relatively low incomes and relatively few medical service resources, and they usually choose buses, subways, or other modes of travel. Therefore, increased medical resources have reduced the demand for taxi pick-up and drop-off among nearby residents. Residents who are further away usually choose to seek medical treatment nearby, and thus will not take taxis. In addition, the positive promotion effect is greater in central Weiyang District. Owing to the relatively abundant medical resources in the region, people often choose convenient and cost-effective transportation such as taxis and private cars when going to buy medicine or see a doctor; a reasonable addition of medical services in this region can increase the total demand for taxi pick-up and drop-off.

*Design dimension*. The regression coefficients of Srn_d on weekdays and weekends are both positive and negative (Figs 8(E) and 9(E)). The overall spatial distributions of the regression coefficients do not differ significantly. Srn_d on weekdays and weekends has a significant positive impact on the total demand for taxi pick-up and drop-off in south-central of Yanta District, most areas of the Beilin District, most areas of the Xincheng District, and south-central and north-central of Weiyang District. These areas have a high population density, and a moderate increase in secondary roads will increase residents’ demand for taxi pick-up and drop-off. The negative inhibitory effect is greater along the West Third Ring Road, Taibai Interchange, and Jinhua South Road. These roads are mostly connected to urban elevated expressways, and subway stations have less radiation; thus, residents are more inclined to travel by private cars.

*Destination accessibility dimension*. The regression coefficients of Dcc on both weekdays and weekends are negative (Figs 8(F) and 9(F)). This shows that with an increase in Dcc, the total demand for taxi pick-up and drop-off decreases. Overall, the magnitude of this decrease gradually increases from east to west and north to south. This is because the southwest part of the central urban area (southwest of Yanta District) has better economic development than the eastern region (northeast, east, and southeast of Baqiao District) and has more shopping centers and office spaces. Residents living in the southwest can choose nearby areas to meet their daily living and work needs. Therefore, Dcc has a greater impact on the choice of taxi travel.

## 6 Conclusions

It is important to investigate the influence of the built environment on taxi travel demand. Previously, little attention has been paid to the MAUP in data aggregation, and the impact of the “5D” dimensions of built environment variables on taxi travel demand was considered less commonly. At the same time, there was a lack of consideration for the nonlinear and linear relationships between the two. This study analyzed the central urban area of Xi’an as an example. A “5D” dimension index system of built environment variables was constructed using multi-source data, which provided a scientific basis for quantifying each index. Based on the OPGD model, an optimal spatial analysis unit was selected to study the effects of the built environment on taxi travel demand, which enriched the quantitative basis for selecting the research units. The OPGD and MGWR models were then cross-used to reveal the influence of the built environment on taxi travel demand from global and local perspectives, thereby providing a method for studying this problem. The main conclusions are summarized as follows:

1. It is necessary to consider the MAUP when discussing the impact of the built environment on taxi travel demand. It is preferable to divide the space units into regular hexagonal grids rather than into square grids. A regular hexagonal grid with a side length of 900m yielded the best effect and was selected as the optimal spatial analysis unit.
2. The factor detection results show that on weekdays or weekends, Dcc, Cr_d, Tf_d, P_d, Cs_d, Ls_d, and Fis_d have a greater influence on the taxi travel demand.
3. Comparing the results of the GWR and MGWR models, the MGWR model considering the spatial heterogeneity scale difference has a better fitting effect. On both weekdays and weekends, the spatial action scale of the Srn_d variable is small. This shows that taxi travel is susceptible to spatial location, and the influence of spatial location on taxi travel should be prioritized in urban construction.
4. The spatial heterogeneity of the regression coefficient shows that Srn_d, Dcc, and Ms_d have a significant influence on local taxi travel demand. Cs_d is significantly influenced by weekdays and weekends, and there is an apparent spatial difference.

These results provide an important reference for urban planners and traffic managers. For urban planners, the optimal spatial analysis unit for taxi travel demand is determined in combination with the built environment, and a demand forecast is conducted on this basis. Centralized passenger carrying points can be set in areas with high demand forecasts. When adjusting the demand for taxis in the entire region, urban planners and traffic managers can prioritize updating and adjusting the built environment variables with greater influence, such as Cr_d, Tf_d, P_d, and Dcc. When making local adjustments to taxi demand, priority can be given to optimizing Srn_d, Dcc, and Ms_d. Improvement strategies can be proposed according to local conditions, optimizing the urban layout, guiding taxi travel behavior, and promoting the virtuous cycle of urban traffic development. For taxi industry management, an optimal spatial analysis unit can provide a reference for traffic zoning in taxi demand forecasting. At the same time, it can guide drivers to areas with a high demand for transportation and promote the scheduling and coordinated development of taxi vehicles.

## 7 Limitations and future work

The above conclusions are expected to help decision-makers formulate targeted urban planning and traffic management strategies. Nevertheless, there are some limitations of this study, and the next steps need to be improved and studied.

1. The variables of an urban built environment are complex and changeable. Although the main elements of the “5D” dimensions are included in the selection of built environment variables, common indicators such as employment density and number of intersections have not been taken into account. In the future, we plan to incorporate these indicators to reveal their relationship with taxi travel demand.
2. The conclusion of this study regarding the selection of an optimal spatial analysis unit is based only on the optimal solution for a specific research area and data set. When changing the study area and data set, the spatial analysis unit must be redefined. However, the determination method is equally applicable to other areas. In the future, we will focus on the calculation of new data in a new study area under various partition schemes, and the effect of the MAUP also needs to be further researched.
3. Although this study discussed the influence of the built environment on taxi travel demand, it did not provide specific suggestions for vehicle scheduling. In the future, a taxi travel demand prediction model will be constructed to provide a reasonable scheme for the dynamic spatial scheduling of taxis.

## Supporting information

S1 Appendix Parameter estimation of the MGWR model. (DOCX)

S2 Appendix Space weight matrix determination of the MGWR model. (DOCX)

S3 Appendix Results of the optimal discretization of built environment variables. (DOCX)
