# Supplementary material for: Influence of the built environment on taxi travel demand based on the optimal spatial analysis unit
Source: PLoS One. 2023 Oct 3;18(10):e0292363. doi: 10.1371/journal.pone.0292363 (PMC10547203; doi:10.1371/journal.pone.0292363)
Supplement: S1 Appendix — (DOCX) [file pone.0292363.s001.docx]

**Parameter estimation of the MGWR model**

The MGWR model uses a back-fitting algorithm to calculate the local regression coefficients of the model, and each local regression coefficient must be preliminarily estimated before starting [1]. Each local regression coefficient is thus initialized and used as an estimate for the MGWR model. There are three initialization methods.

- Use the estimated values of the GWR model for initialization.
- Use the estimated values of the OLS model for initialization.
- Set all parameter estimates to zero.

Using the estimated value of the GWR model for initialization can significantly reduce the number of iterations and obtain a higher bandwidth optimization efficiency; therefore, this method was chosen in this study to obtain the initialization parameter, $\beta_{h_{ik}}(\mu_{i},\nu_{i})$. Multiplying this by the independent variable $x_{ik}$ yields the following:

| $f_{ik}=\beta_{ik}(\mu_{i},\nu_{i})x_{ik}$ | (1) |
| --- | --- |

At this point, the formula for the MGWR model becomes:

| $y_{i}=\beta_{i0}(\mu_{i},\nu_{i})+\sum_{k=1}^{m} f_{ik}+\varepsilon_{i}$ $i=1,2,3,...,n$ | (2) |
| --- | --- |

We then calculate the difference between the observed value and the fitting value obtained from the initial estimate to obtain the initialization error, $\widehat{\varepsilon_{i}}$:

| $\widehat{\varepsilon_{i}}=y_{i}-\sum_{k=1}^{m} \widehat{f}_{ik}$ | (3) |
| --- | --- |

Furthermore, the initial error, $\widehat{\varepsilon_{i}}$, is added to $\sum_{k=1}^{m} f_{ik}$ and then calibrated with the independent variable $x_{ik}$ using a geographically weighted regression; the calibration is performed iteratively. At each iteration, the optimal bandwidth is determined, new local parameters are estimated, and the previous estimates are replaced. By analogy, until the iteration terminates, the difference in the parameter estimates for successive iterations converges to a specified threshold. There are two common criteria for evaluating whether the difference in parameter estimation converges.

(1) $SOC_{RSS}$

$SOC_{RSS}$ refers to the maximum difference between the sum of the squared residuals of the two regression steps that does not exceed the convergence value. The calculation formula is as follows:

| $SOC_{RSS}=ABS(RSS_{new}-RSS_{old})/RSS_{new}$ | (4) |
| --- | --- |

where $RSS_{new}$ is the sum of the squares of the residuals, $RSS_{old}$ is the sum of the squares of the residuals from the previous step, and ABS is the absolute value function.

(2) $SOC_{\text{f}}$

$SOC_{f}$ refers to the maximum difference between the regression coefficients of the previous step and those of the current step that does not exceed the convergence value. The calculation formula is as follows:

| $SOC_{f}=SQRT[\sum_{j=1}^{p} \sum_{i=1}^{n} (\widehat{f}_{ijk}^{new}-\widehat{f}_{ijk}^{old})^{2}/\sum_{i=1}^{n} (\sum_{j=1}^{p} \widehat{f}_{ijk}^{new})^{2}]$ | (5) |
| --- | --- |

where $\widehat{f}_{ijk}^{old}$ is the product of the regression coefficient of the previous step and the independent variable. $\widehat{f}_{ijk}^{new}$ is the product of the regression coefficient in the current step and the independent variable, and $SQRT$ is the square root function.

It should be noted that $SOC_{RSS}$ is concerned with the fitting degree of the whole model, while $SOC_{f}$ is concerned with the fitting degree of the new relation. However, for the MGWR model, $SOC_{f}$ is more consistent with the demand for model optimization.

**References**

1. Cao Y, Tian YZ, Tian JL, Liu KN, Wang Y. Impact of built environment on residential online car-hailing trips: Based on MGWR model. Plos One. 2022;17(11). doi: 10.1371/journal.pone.0277776.
