# Supplementary material for: Influence of the built environment on taxi travel demand based on the optimal spatial analysis unit
Source: PLoS One. 2023 Oct 3;18(10):e0292363. doi: 10.1371/journal.pone.0292363 (PMC10547203; doi:10.1371/journal.pone.0292363)
Supplement: S2 Appendix — (DOCX) [file pone.0292363.s002.docx]

**Space weight matrix determination of the MGWR model**

Determining the space weight matrix of the MGWR model primarily includes three aspects: the type of spatial distance measurement, type of spatial weight function, and bandwidth selection criteria. Here, the most commonly used Euclidean distance is chosen for the spatial distance calculation. The calculation formula is as follows:

| $d_{ij}=\sqrt{(\mu_{i}-\mu_{j})^{2}+(\nu_{i}-\nu_{j})^{2}}$ | (1) |
| --- | --- |

where $(\mu_{i},\nu_{i})$ represents the geographical coordinates of the centroid of the $ith$ grid, $(\mu_{j},\nu_{j})$ represents the geographical coordinates of the centroid of the $jth$ grid, and $d_{ij}$ is the distance between the centroids of the $ith$ and $jth$ grids.

The space weight function is an the adaptive bi-square kernel function, which can select different bandwidths according to itself. The calculation formula is as follows:

|  | (2) |
| --- | --- |

where $w_{ij}$ is the space weight function between the centroids of the $ith$ and $jth$ grids, $h$ is the bandwidth, $d_{ij}$ is the distance between the centroids of the $ith$ and $jth$ grids, and $h_{i(k)}$ is the adaptive bandwidth, representing the optimal bandwidth of $k$ adjacent points of the $ith$ grid centroid.

The bandwidth selection criterion adopted is the Akaike information criterion (AIC). The AIC is a model selection criterion proposed by Akaike in 1974 to measure the goodness-of-fit of statistical models [1], also known as the minimum information criterion. It has a reasonable degree of optimization and is widely used because of its simplicity and convenience. The calculation formula is as follows:

| $AIC=-2ln(L)+2k$ | (3) |
| --- | --- |

where $L$ is the maximum-likelihood function, and $k$ represents the number of independent variables in the regression model. The smaller the value of $AIC$, the better the corresponding model will be.

Later, Brunsdon et al. [2] improved the AIC and proposed a modified Akaike Information Criterion (AICc) applicable to the GWR model. The calculation formula is as follows:

| $AICc=-2nln(\widehat{\sigma})+n\ln(2\pi)+n\frac{n+tr(s)}{n-2-tr(s)}$ | (4) |
| --- | --- |
| $\widehat{\sigma}=\frac{RSS}{n-tr(s)}$ | (5) |

where $n$ denotes the total number of research units, $\widehat{\sigma}$ is the maximum likelihood estimate of the variance in the random error term, and $tr(s)$ is a trace of hat matrix $S$ in the GWR model. The smaller the value of $AICc$, the better the corresponding model will be.

**References**

1. Akaike H. A new look at the statistical model identification. IEEE Trans Autom Control (USA). 1974;AC-19(6):716-23. doi: 10.1109/tac.1974.1100705.

2. Brunsdon C, Fotheringham AS, Charlton M. Geographically weighted summary statisticsa framework for localised exploratory data analysis. Comput Environ Urban Syst (UK). 2002;26(6):501-24. doi: 10.1016/s0198-9715(01)00009-6.
