# Supplementary material for: Influence of the built environment on taxi travel demand based on the optimal spatial analysis unit
Source: PLoS One. 2023 Oct 3;18(10):e0292363. doi: 10.1371/journal.pone.0292363 (PMC10547203; doi:10.1371/journal.pone.0292363)
Supplement: S3 Appendix — (DOCX) [file pone.0292363.s003.docx]

**Results of the optimal discretization of built environment variables**

**Table 1. The optimal discretization results of built environment variables at 100-500m scale (Regular hexagonal grid).**

| Scale | 100m | | 200m | | 300m | | 400m | | 500m | |
| --- | --- | --- | --- | --- | --- | --- | --- | --- | --- | --- |
| Built environment variables | Optimal discretization method | Classification number | Optimal discretization method | Classification number | Optimal discretization method | Classification number | Optimal discretization method | Classification number | Optimal discretization method | Classification number |
| P_d | geometric | 10 | geometric | 10 | geometric | 10 | natural | 9 | natural | 9 |
| Cs_d | sd | 10 | sd | 10 | quantile | 9 | quantile | 10 | quantile | 10 |
| [Ta_](javascript:;)d | quantile | 5 | quantile | 8 | quantile | 8 | quantile | 5 | quantile | 10 |
| Pa_d | quantile | 6 | quantile | 7 | quantile | 4 | quantile | 5 | sd | 4 |
| Ss_d | sd | 10 | sd | 10 | sd | 10 | quantile | 7 | quantile | 8 |
| Tf_d | natural | 10 | sd | 4 | sd | 10 | quantile | 8 | sd | 10 |
| Et_d | quantile | 6 | sd | 5 | sd | 10 | sd | 10 | sd | 10 |
| Fis_d | quantile | 6 | sd | 4 | quantile | 7 | quantile | 9 | sd | 10 |
| Cr_d | quantile | 7 | sd | 4 | sd | 7 | sd | 10 | sd | 10 |
| Ls_d | sd | 10 | sd | 10 | quantile | 10 | quantile | 10 | quantile | 9 |
| Sls_d | quantile | 7 | sd | 4 | quantile | 9 | sd | 10 | sd | 10 |
| [Ms_](javascript:;)d | natural | 8 | sd | 4 | sd | 10 | sd | 10 | sd | 10 |
| Gaso_d | quantile | 9 | sd | 4 | quantile | 8 | sd | 10 | sd | 10 |
| As_d | quantile | 7 | quantile | 10 | sd | 10 | sd | 10 | quantile | 10 |
| Mduf | quantile | 10 | quantile | 9 | equal | 10 | equal | 9 | quantile | 8 |
| Prn_d | quantile | 8 | sd | 10 | sd | 10 | natural | 8 | sd | 10 |
| Srn_d | quantile | 9 | quantile | 9 | quantile | 10 | quantile | 9 | quantile | 8 |
| Trn_d | quantile | 10 | quantile | 9 | natural | 9 | sd | 10 | quantile | 8 |
| Uern_d | natural | 8 | sd | 10 | natural | 9 | natural | 9 | natural | 9 |
| Dcc | sd | 9 | geometric | 10 | sd | 10 | sd | 9 | geometric | 9 |
| Bs_d | quantile | 4 | quantile | 6 | quantile | 8 | natural | 4 | quantile | 9 |
| Ss_d | quantile | 3 | quantile | 3 | geometric | 3 | equal | 3 | quantile | 3 |

**Table 2. The optimal discretization results of built environment variables at 600-1000m scale (Regular hexagonal grid).**

| Scale | 600m | | 700m | | 800m | | 900m | | 1000m | |
| --- | --- | --- | --- | --- | --- | --- | --- | --- | --- | --- |
| Built environment variables | Optimal discretization method | Classification number | Optimal discretization method | Classification number | Optimal discretization method | Classification number | Optimal discretization method | Classification number | Optimal discretization method | Classification number |
| P_d | natural | 10 | natural | 10 | natural | 10 | natural | 10 | sd | 10 |
| Cs_d | quantile | 10 | quantile | 10 | quantile | 9 | quantile | 7 | quantile | 7 |
| [Ta_](javascript:;)d | sd | 10 | quantile | 9 | sd | 10 | sd | 7 | quantile | 10 |
| Pa_d | sd | 10 | sd | 10 | sd | 10 | sd | 10 | sd | 10 |
| Ss_d | quantile | 10 | quantile | 10 | quantile | 10 | quantile | 10 | quantile | 10 |
| Tf_d | natural | 10 | quantile | 6 | natural | 8 | natural | 10 | natural | 10 |
| Et_d | sd | 10 | quantile | 10 | sd | 10 | natural | 9 | natural | 10 |
| Fis_d | sd | 10 | quantile | 10 | sd | 10 | sd | 10 | sd | 10 |
| Cr_d | sd | 10 | quantile | 10 | sd | 10 | natural | 10 | natural | 10 |
| Ls_d | quantile | 9 | quantile | 10 | quantile | 6 | quantile | 10 | quantile | 10 |
| Sls_d | sd | 10 | sd | 10 | quantile | 9 | sd | 10 | natural | 10 |
| [Ms_](javascript:;)d | sd | 10 | sd | 10 | quantile | 8 | quantile | 9 | quantile | 9 |
| Gaso_d | sd | 10 | sd | 10 | sd | 10 | natural | 9 | sd | 10 |
| As_d | quantile | 10 | sd | 10 | sd | 10 | quantile | 10 | quantile | 9 |
| Mduf | natural | 10 | quantile | 8 | natural | 7 | quantile | 9 | equal | 10 |
| Prn_d | quantile | 10 | quantile | 9 | sd | 10 | natural | 10 | natural | 9 |
| Srn_d | sd | 10 | natural | 8 | sd | 10 | sd | 10 | sd | 10 |
| Trn_d | natural | 8 | quantile | 10 | natural | 10 | sd | 10 | quantile | 10 |
| Uern_d | quantile | 9 | natural | 9 | sd | 10 | natural | 8 | sd | 10 |
| Dcc | sd | 10 | sd | 10 | sd | 10 | sd | 9 | sd | 9 |
| Bs_d | equal | 10 | quantile | 10 | sd | 8 | sd | 5 | sd | 9 |
| Ss_d | equal | 3 | geometric | 3 | geometric | 3 | quantile | 3 | geometric | 3 |

**Table 3. The optimal discretization results of built environment variables at 100-500m scale (Square grid)**

| Scale | 100m | | 200m | | 300m | | 400m | | 500m | |
| --- | --- | --- | --- | --- | --- | --- | --- | --- | --- | --- |
| Built environment variables | Optimal discretization method | Classification number | Optimal discretization method | Classification number | Optimal discretization method | Classification number | Optimal discretization method | Classification number | Optimal discretization method | Classification number |
| P_d | geometric | 10 | geometric | 10 | geometric | 10 | geometric | 10 | geometric | 10 |
| Cs_d | sd | 10 | sd | 10 | quantile | 8 | quantile | 10 | quantile | 10 |
| [Ta_](javascript:;)d | quantile | 5 | natural | 7 | quantile | 10 | quantile | 10 | quantile | 9 |
| Pa_d | quantile | 4 | quantile | 6 | quantile | 6 | quantile | 9 | quantile | 10 |
| Ss_d | sd | 10 | quantile | 10 | quantile | 9 | quantile | 8 | quantile | 9 |
| Tf_d | quantile | 10 | sd | 4 | sd | 10 | natural | 9 | quantile | 6 |
| Et_d | natural | 10 | sd | 5 | sd | 10 | sd | 10 | sd | 10 |
| Fis_d | quantile | 10 | quantile | 8 | quantile | 9 | sd | 10 | sd | 10 |
| Cr_d | quantile | 7 | sd | 4 | sd | 10 | sd | 10 | sd | 10 |
| Ls_d | sd | 10 | sd | 10 | quantile | 8 | quantile | 9 | quantile | 7 |
| Sls_d | quantile | 8 | quantile | 9 | sd | 4 | sd | 10 | sd | 10 |
| [Ms_](javascript:;)d | natural | 7 | quantile | 6 | sd | 10 | sd | 10 | sd | 10 |
| Gaso_d | quantile | 10 | sd | 4 | sd | 4 | sd | 10 | sd | 10 |
| As_d | quantile | 5 | sd | 10 | sd | 10 | sd | 10 | quantile | 10 |
| Mduf | natural | 10 | natural | 10 | equal | 9 | geometric | 10 | quantile | 9 |
| Prn_d | quantile | 9 | natural | 9 | quantile | 10 | quantile | 10 | quantile | 8 |
| Srn_d | quantile | 10 | quantile | 9 | quantile | 10 | quantile | 10 | quantile | 6 |
| Trn_d | quantile | 10 | quantile | 8 | quantile | 8 | quantile | 9 | quantile | 9 |
| Uern_d | natural | 10 | natural | 9 | natural | 8 | quantile | 9 | quantile | 10 |
| Dcc | sd | 9 | sd | 9 | sd | 9 | sd | 10 | sd | 10 |
| Bs_d | geometric | 7 | quantile | 6 | quantile | 7 | geometric | 9 | quantile | 10 |
| Ss_d | equal | 3 | equal | 3 | equal | 3 | equal | 3 | quantile | 3 |

**Table 4. The optimal discretization results of built environment variables at 600-1000m scale (Square grid)**

| Scale | 600m | | 700m | | 800m | | 900m | | 1000m | |
| --- | --- | --- | --- | --- | --- | --- | --- | --- | --- | --- |
| Built environment variables | Optimal discretization method | Classification number | Optimal discretization method | Classification number | Optimal discretization method | Classification number | Optimal discretization method | Classification number | Optimal discretization method | Classification number |
| P_d | natural | 10 | equal | 9 | natural | 10 | natural | 10 | sd | 10 |
| Cs_d | quantile | 9 | quantile | 10 | quantile | 8 | quantile | 10 | quantile | 9 |
| [Ta_](javascript:;)d | sd | 10 | sd | 10 | quantile | 9 | sd | 10 | sd | 10 |
| Pa_d | sd | 10 | sd | 10 | sd | 10 | sd | 10 | sd | 10 |
| Ss_d | quantile | 10 | quantile | 10 | quantile | 9 | quantile | 10 | quantile | 7 |
| Tf_d | quantile | 8 | natural | 10 | sd | 10 | quantile | 8 | natural | 10 |
| Et_d | quantile | 9 | quantile | 10 | sd | 10 | quantile | 10 | natural | 9 |
| Fis_d | sd | 10 | sd | 10 | quantile | 8 | sd | 10 | sd | 10 |
| Cr_d | sd | 10 | natural | 10 | sd | 10 | sd | 10 | quantile | 10 |
| Ls_d | quantile | 7 | quantile | 9 | quantile | 9 | quantile | 10 | natural | 10 |
| Sls_d | sd | 10 | sd | 10 | quantile | 10 | natural | 10 | natural | 10 |
| [Ms_](javascript:;)d | sd | 10 | sd | 10 | sd | 10 | quantile | 8 | quantile | 10 |
| Gaso_d | sd | 10 | natural | 9 | sd | 10 | sd | 10 | natural | 10 |
| As_d | sd | 10 | sd | 10 | quantile | 10 | sd | 10 | quantile | 9 |
| Mduf | quantile | 8 | quantile | 10 | natural | 8 | natural | 10 | natural | 8 |
| Prn_d | quantile | 10 | quantile | 9 | quantile | 8 | quantile | 10 | quantile | 9 |
| Srn_d | quantile | 8 | quantile | 8 | quantile | 9 | quantile | 7 | quantile | 9 |
| Trn_d | quantile | 7 | quantile | 10 | quantile | 10 | natural | 9 | quantile | 7 |
| Uern_d | quantile | 9 | natural | 10 | sd | 10 | quantile | 10 | sd | 10 |
| Dcc | sd | 9 | sd | 10 | sd | 10 | sd | 9 | sd | 9 |
| Bs_d | quantile | 10 | sd | 5 | sd | 5 | sd | 10 | sd | 10 |
| Ss_d | geometric | 3 | quantile | 3 | geometric | 3 | equal | 3 | quantile | 3 |
